# Managing Shiga Toxin-Producing *E. coli* Using Statistical Process Control Charts for Routine Health and Production Monitoring in Pig Farming

**DOI:** 10.3389/fvets.2022.814862

**Published:** 2022-03-17

**Authors:** Annalisa Scollo, Mattia Fasso, Patrizia Nebbia, Claudio Mazzoni, Claudia Cossettini

**Affiliations:** ^1^Department of Veterinary Sciences, University of Torino, Torino, Italy; ^2^Swivet Research sas, Reggio Emilia, Italy; ^3^Chemifarma spa, Forlì, Italy

**Keywords:** Shiga-toxin, *Escherichia coli*, Oedema disease, pig, statistical process control (SPC)

## Abstract

Oedema disease (ED) caused by Shiga-toxin-producing *E. coli* in pigs is a serious life-threatening disease, particularly among weaned piglets. When a preventive protocol is adopted in a specific farm, interpretation of effectiveness is often complicated in field conditions due to natural or “common cause” variation. For this reason, in this study a Statistical process control (SPC) approach was used to retrospectively evaluate the application of an ED preventive protocol (lower protein diet, *ad-libitum* fiber, vaccination at 5 days of age) in an infected commercial piglets' weaning site. The analysis was established over a 9-years period (*n* = 75 consecutive batches; 1,800 weaners per batch) using mortality for each batch as the key parameter of health and production; the statistics and the control limits (mean ± 3-fold sd; UCL, upper control limit; LCL, lower control limit) were based on data from the first 28 batches (Period 1) before the onset of the first ED clinical signs. The charts allowed the detection of defined out of control batches (i.e., with mortality out of the intervention limits) from batch 29 ongoing, exploring a Period 2 (unstable production and ED clinical signs; 36 batches) and a Period 3 (application of the ED preventive protocol; 11 batches). Mortality evaluation using SPC revealed a production system defined under-control (mean moving range bar = 1,34%; UCL = 4,37%; LCL = 0%) during Period 1. During Period 2, charts lost the state of statistical control, as showed by several signals of special cause variation due to the ED outbreak. Period 3 was characterized again by a state of statistical control, where no signals of special cause variation was showed. In conclusion, the retrospective application of SPC charts in the present study was able to confirm the efficacy of an ED preventive protocol in reducing mortality in a piglets' weaning site. SPC charting is suggested as an useful tool to provide insights into relationships between health, managerial, and welfare decision and some selected iceberg parameters in livestock.

## Introduction

Oedema disease (ED) in pigs is caused by Shiga-toxin-producing *E. coli* (STEC), also known as Oedema disease *E. coli*. These strains of *E. coli* are characterized by the ability to produce the Shiga toxin 2e (Stx2e), which enters the bloodstream and damages vessel walls resulting in oedema in targeted tissues, causing a serious life-threatening disease particularly among weaned piglets. Animals suffering from ED show oedema, emaciation, neurological disorders such as ataxia or paralysis, and sudden death in severe cases (1). Once a farm becomes infected, it is generally difficult to exclude ED from the pigpen and the same strain is usually found in many consecutives batches of pigs; this may result in great damages to the pig raisers (2). When piglets show symptoms of ED, antibiotic treatment is still the most widely used therapeutic approach to improve their health condition, but it is often late to rescue them as the toxin already spreads throughout the body by then. However, prophylactic use of antibiotics on other healthy piglets or as food additive to reduce colonization of pathogenic bacteria in the gut is controversial since it is known to increase drug-resistant bacteria, which is of great concern in pig farms (3, 4). The contribution of antibiotics to the potential development of antibiotic-resistant strains of bacteria (5) prompted the European Union (EU) to implement a full ban on their use as growth promoters in livestock in January 2006 (Regulation 1831/2003/EC on additives for use in animal nutrition). This measure was first applied in Sweden and Denmark, leading to an important increase in the prevalence of Post-weaning diarrhea and mortality rates due to *Escherichia coli* infections (6). In addition, colistin (a polymyxin) is considered one of the most effective antimicrobials in the treatment of *E. coli* infection in pigs, but it is considered one of the three classes of antimicrobial agents listed in the WHO list (2019) of critically important antimicrobials (fluoroquinolones, polymyxins and third- or fourth-generation cephalosporins) most urgently requiring management of the risks from antimicrobial resistance. Another alternative for controlling *E. coli* infections has been the use of in-feed zinc oxide (ZnO), but the European Commission has finally decided to ban the therapeutic use of ZnO in feed by 2022 due to its contribution to the potential increase of antimicrobial resistance and environmental issues (7). For these reasons, the development of an effective preventive approach is awaited. The Food and Agriculture Organization (FAO) has also emphasized the need to prevent infectious diseases in animals through several measures compiled into three main categories: good animal husbandry, effective biosecurity, and vaccination. For example, some feed management strategies, such as restriction of feed intake, reduction of crude protein and digestible energy, and high fiber diets have also been reported as effective in controlling *E. coli* infection outbreaks (8).

When a preventive protocol is adopted in a specific farm, it is essential to monitor its effectiveness on the pig health and production process. Unfortunately, interpretation of effectiveness is often complicated in field conditions due to natural or “common cause” variation (9). Statistical process control (SPC) charts, invented in the 1920s and used in industry for many years, provide a statistical approach that might be useful also in human healthcare (10–13) and animal production (14–18).

Important tools for the SPC include control charts, such as the individual value chart (I-chart) and the moving range chart (MR-chart). An SPC chart is a chronological graph of process data (i.e., the parameter of interest regularly recorded during time) with a center line (usually the mean) and upper and lower control limits defined statistically (10–13, 19). If all process values fall between the upper and lower limits, the process is considered in “statistical control”. If process values fall outside the limits, or exhibit a particular trend of variation (e.g., progressive increasing or decreasing values from the center line), this provides evidence of a “special” cause of variation (19). SPC charts of historical data can determine whether a process has been in “statistical control”, can be used prospectively to detect process changes after the introduction of a new procedure in the productive chain and its effectiveness and, when a chart indicates the establishment of a changed level of performance, to calculate a new center line and limits. The I-chart and MR-chart are commonly used in case of data that are continuous and not collected in subgroups. The I-chart displays the individual data and monitors mean and shift in the process while the variation is monitored by the MR-chart (14). In human healthcare, SPC charts have been used to discriminate between changes that yield improvement and those that do not, by visualization and analysis of the performance of a process over time [including biological processes such as blood pressure homoeostasis or organizational processes such as patient care in a hospital; (20, 21)], sometimes in real time. In the swine sector, SPC charts were used to reveal changes in a production process after Porcine Reproductive and Respiratory Syndrome outbreak or vaccination (22, 23). Statistically derived decision rules help users to determine whether the performance of a process is stable and predictable or whether there is variation in the performance that makes the process unstable and unpredictable, influencing the decision-making phase. One source of such variation can be a successful intervention aimed at improvement that changes performance for the better. If the improvement is maintained, the process will stabilize again at its new level of performance (24).

In the present study, data on mortality in a piglets weaning site have been retrospectively control-charted during a 9-years production process, with the aims to: (I) apply SPC charts in livestock as a monitoring tool of pig health through a key parameter; (II) verify the effectiveness of a preventive protocol for ED during time.

## Materials and Methods

### Animals and Facilities

The study took place in a commercial weaning site located in the Italian region Lombardy. The 1,200-sows farrowing site producing piglets for the weaning site was of the same owner, located 4 km far, and organized in a 3-week batch system, following all-in all-out procedures. Piglets were weaned at 4 weeks of age, moved to the weaning site every 6 weeks (one batch in, one batch to other sites) and housed in the nursery facilities for 11 weeks. The nursery barn included 2 identical sectors completely separated, with 8 identical rooms containing 8 pens each; each sector was allotted to one batch and managed with all-in all-out procedures. Environmental parameters in the nursery barn were set up according to piglet needs. During the entire study, the farm was positive but stable to porcine reproductive and respiratory syndrome virus (PRRSV) infection, positive to Mycoplasma hyopneumoniae, and positive to Porcine circovirus 2 (PCV2) but without evidence of clinical expression. Piglets were vaccinated for Mycoplasma hyopneumoniae and PCV2. Animals were fed *ad libitum* with dry feed.

### Data Collection

From January 2013 to August 2021, dead animals were registered for each batch. After the first manual recording of the mortality directly by a trained stockman on a register in the farm, a formal verification was systematically made by the production office, that verified the number of sold animals on the total pigs arrived for each batch.

### ED Status, Feed Strategies and Interventions

#### Period 1: Production in Control

From January 2013 to May 2016 (28 batches) the farm did not show any clinical sign related to STEC infection. The piglets received a commercial feed that included ZnO (2,500 ppm) for the first 14 days. The amount of crude protein in the starting feed was 21%, with 5,5% of fiber.

#### Period 2: Unstable Production and ED Clinical Signs

The weaning site started to show clinical signs of ED from June 2016 (36 batches). The disease was diagnosed based on clinical signs (nervous signs and sudden death), anatomopathological lesions (gelatinous oedema in the gastric cardia, mesocolon, small intestinal mesentery and gallbladder) and laboratory analyses. STEC infection was identified by detection of genes coding for virulence factors Stx2e and fimbriae F18 by qPCR multiplex (Istituto Zooprofilattico Sperimentale della Lombardia e dell'Emilia Romagna, Italy) from jejunum content samples. The piglets still received the same commercial feed of period 1. As suggested by the antibiogram, gentamicin (4 mg/kg body weight) was administered for 5 days in drinking water after each batch accommodation. In case of poor clinical recovery, other antibiotic treatments were administered without a fixed protocol of selection (colistin or apramycin, both sensible to antibiogram).

#### Period 3: The ED Preventive Protocol

From May 2020 to the day of the data analysis (11 batches), the farm adopted a preventive protocol for ED: (I) The piglets received a commercial feed that still included ZnO (2,500 ppm) for the first 14 days, but with a lower amount of crude protein (17%); (II) A continuous provision of fiber through long straw in a rack was furnished in all the pens; (III) Animals were vaccinated against ED (Ecoporc SHIGA®, CEVA Salute Animale, Agrate Brianza, Italy) in the farrowing room. The dosage applied to piglets was a single intramuscular injection (1 mL) of a genetically modified recombinant Stx2e antigen for the active immunization of piglets from the age of 4 days onwards (25).

### Creation of Charts

Data on mortality for each batch were entered onto an Excel sheet. SPC charts were produced using SPC IV Excel (Quality America, Inc.). Mortality as the process performance indicator was the proportions (as percentages) of piglets died in each specific batch. Following the Wheeler and Poling's (26) advice, Individuals and Moving Range charts (I-MR charts) were used. The individual chart displays individual measurements. The moving range chart shows the absolute value of the difference between consecutive measurements. On an I-MR chart the center line (process center line, PCL) represents the mean of the values used for computation of the control limits, calculated by the following formula:


$$\begin{array}{l} \bar{X}= \end{array}\frac{\sum_{i=1}^{k}xi}{k}$$

Where, xi = Value at point i; k = Number of individual data.

Upper warning limits (UWL) and lower warning limits (LWL) were calculated using two standard deviations:


$$\begin{array}{l} UWL=\;\bar{X}+2\sigma \\ LWL\;=\;\bar{X}-\;2\sigma \end{array}$$

Where σ = Process standard deviation.

When UWL and LWL are exceeded, it serves as an indication that a process is changing and in need of attention (warning signal). Upper control limits (UCL) and lower control limits (LCL) were calculated using three standard deviations above and below the center line and, when exceeded, the process is dramatically considered out of control (27).


$$\begin{array}{l} UCL=\;\bar{X}+3\sigma \\ LCL\;=\;\bar{X}-\;3\sigma \end{array}$$

The standard deviation was estimated using the mean moving range. Mean MR is the average of all range value which is calculated by the following equation:


$$\begin{array}{l} \bar{MR}=\frac{\sum_{i=2}^{k}\;\left|x_{i}-x_{i-1}\right|}{k-1} \end{array}$$

Procedures for setting limits in the I-MR charts were those suggested by Sanghangthum et al. (27): the goal is to ensure that the process operating in a state of statistical control, which means the process is predictable within the limits determined using data from the process. The MR chart of the 28 observations from Period 1 yielded no signals of special cause variation, so Period 1 was defined as the in-control reference period and it was used to set limits in the analysis following suggestion of Sanghangthum et al. (27), that recommended to collect at least 10–20 in-control data points for retrospective process analysis; the center line and limits based on these observations to monitor performance were then extended also in Period 2 and 3. The software labels relevant points with the test number that signals evidence of special cause variation (28). Average mortality (%) during each of the three periods was also calculated by a descriptive analysis.

All eight tests for special cause variation listed below and available in SPC IV Excel were applied:

Test 1: 1 point beyond 3 standard deviations (out of control signal).
Test 2: 9 successive points same side of PCL (warning signal).
Test 3: 6 successive points increasing or decreasing (warning signal).
Test 4: 14 successive points alternating up and down (warning signal).
Test 5: 2 out of 3 successive points beyond 2 standard deviations (same side, warning signal).
Test 6: 4 out of 5 successive points beyond 1 standard deviation (same side, warning signal).
Test 7: 15 successive points within 1 standard deviation (either side, warning signal).
Test 8: 8 successive points not within 1 standard deviation (either side, warning signal).

## Results

**Figure 1:**
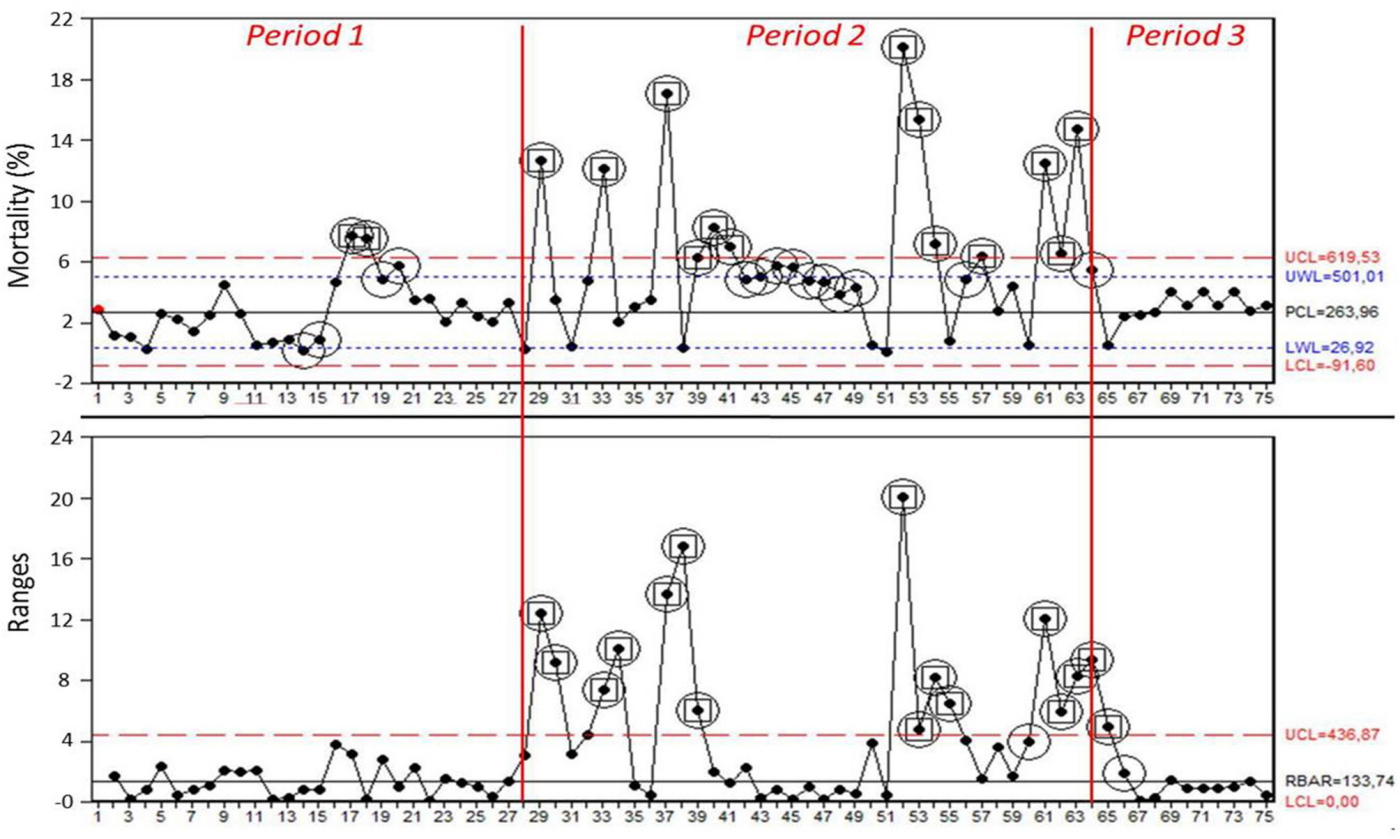
I-MR charts for mortality (%) in each batch obtained from the analysis of the 3 Periods (Period 1 = 1–28; Period 2 = 29–63; Period 3 = 64–75). Process center line (PCL, black line), upper control limit and lower control limit (UCL, LCL, red dotted lines), and upper warning limit and lower warning limit (UWL, LWL, blue dotted lines) were calculated based on observations of Period 1. Circled points showed at least one signal of special cause variation, as expressed by software tests. Circled and framed points showed to be out of control in the process.

Data on mortality in the weaning site were collected for 75 batches of piglets. The mean mortality was 2,26% in Period 1, 5,54% in Period 2, and 3,32% in Period 3. Figure 1 showed I-MR charts where the 28 observations from Period 1 were used to set the center line and limits used also in Period 2 and 3: circled points showed a warning signal of special cause variation; circled and framed points showed to be out of control in the process. Details about both warning signals of special cause variation and points out of control in the process are reported in Table 1. The part of the MR chart showing the 28 observations from the Period 1 suggested that the process operated in a state of statistical control (mean range bar RBAR = 1,34%; UCL = 4,37%; LCL = 0%); I chart of the same Period (PCL = 2,26%; UCL = 5,81%; LCL = −1,30%) yielded two warning signals of special cause variation via test 6, followed by further signals via tests 1, 5, 6, 8 on four observations from batch 17 onwards (Table 1). Frequency of out of control signals in I chart was 7,14%. As no signals of special cause variation emerged in the MR chart in Period 1, the center line and limits based on these observations were used to extend the chart to monitor performance also in Period 2 and 3 (21). From batch 29 onwards (Period 2), MR chart lost the state of statistical control, as showed by several signals of special cause variation, reflected by the same number of positive tests in the I chart (Table 1). Frequency of out of control signals in I chart was 36,11%. Period 3 was characterized by a new state of statistical control yielded no signals of special cause variation from batch 67 onwards. Frequency of out of control signals in I chart was 0%.

**Table 1:** List of special cause variations (run tests) for mortality (%) in each batch related to the I-MR chart obtained from the analysis of the 3 Periods (Period 1 = 1–28; Period 2 = 29–63; Period 3 = 64–75).

**Batch**	Run test(s)^*^		**Batch**	Run test(s)^*^	
	**I chart**	**MR chart**		**I chart**	**MR chart**
1	–	–	39	1, 5^**^	1^**^
2	–	–	40	1, 5^**^	–
3	–	–	41	1, 5, 6^**^	–
4	–	–	42	5, 6	–
5	–	–	43	5, 6	–
6	–	–	44	5, 6, 8	–
7	–	–	45	5, 6, 8	–
8	–	–	46	5, 6, 8	–
9	–	–	47	2, 6, 8	–
10	–	–	48	2, 6, 8	–
11	–	–	49	2, 6, 8	–
12	–	–	50	8	–
13	–	–	51	8	–
14	6	–	52	1, 8^**^	1^**^
15	6	–	53	1, 5, 8^**^	1^**^
16	–	–	54	1, 5, 8^**^	1, 4^**^
17	1^**^	–	55	8	1, 4^**^
18	1, 5, 8^**^	–	56	5, 6, 8	–
19	5, 6, 8	–	57	1, 5, 6, 8^**^	–
20	5, 6, 8	–	58	–	–
21	–	–	59	–	–
22	–	–	60	–	2
23	–	–	61	1^**^	1, 2^**^
24	–	–	62	1, 5^**^	1, 2^**^
25	–	–	63	1, 5, 6^**^	1, 2^**^
26	–	–	64	5, 6	1, 2^**^
27	–	–	65	–	1, 2^**^
28	–	–	66	–	2
29	1^**^	1^**^	67	–	–
30	–	1^**^	68	–	–
31	–	–	69	–	–
32	–	–	70	–	–
33	1, 5, 6^**^	1^**^	71	–	–
34	–	1^**^	72	–	–
35	–	–	73	–	–
36	–	–	74	2	–
37	1^**^	1^**^	75	2	–
38	–	1^**^			

*Process center line (PCL), upper control limit (UCL) and lower control limit (LCL) were calculated based on observations of Period 1*.

* *Test 1: 1 point beyond 3 standard deviations; Test 2: 9 successive points same side of PCL; Test 3: 6 successive points increasing or decreasing; Test 4: 14 successive points alternating up and down; Test 5: 2 out of 3 successive points beyond 2 standard deviations (same side); Test 6: 4 out of 5 successive points beyond 1 standard deviation (same side); Test 7: 15 successive points within 1 standard deviation (either side); Test 8: 8 successive points not within 1 standard deviation (either side)*.

** *Process out of control (more than 3 standard deviations from the PCL)*.

## Discussion

Oedema disease caused by Stx2e-producing strains of *E. coli* can be a significant economic disorder in a pig production farm (29). The cost of associated outbreaks mostly depends on the mortality rate, in addition to weight of the pigs that die (the older the pigs, the more expensive the consequences). Moreover, a possible immunosuppressive effect of the Stx2e toxin is also suspected, which may affect whether piglets thrive properly. Vaccination and diet intervention (30, 31) are considered the main alternatives for protecting the piglets against this disease, avoiding the use of antimicrobials that may trigger the selection for antimicrobial resistance (32). In this study, a vaccine against Stx2e and a reduction from 21 to 17% of crude proteins in the diet were used in a farm where STEC was previously confirmed. Data on mortality have been collected for 9 years since January 2013, during which the farm experienced three different statuses: production defined in control (Period 1), unstable production and ED clinical signs (Period 2), and ED preventive protocol application (Period 3). During Period 1 there were few signals of special cause variation in mortality, 2 out of 6 indicating improvements in the production process, as showed by 5 successive batches with an average mortality lower than 1 standard deviation from the mean. The other 4 signals of special cause variation were unexpected and impossible to address to an underlying reason due to the long time between data collection and analysis, but their variation did not negatively influence the process analysis as confirmed by the MR chart that remained in-control. In June 2016, when the farm started to show clinical signs related to ED, average mortality increased from 2,26 to 5,54%. Besides numerous batches showing a severe mortality with peaks greatly over the UCL and several warning signals of worsening of the production process (8 consecutive batches with an average mortality >1 standard deviation from the mean), an adjunctive important indicator of system out of control was the persistent and exceptional variation in mortality batch after batch showed by the MR-chart, and the increase of out of control signals from 7,14 to 36,11%. The huge fluctuation in mortality that characterized the Period 2 might confirm the various and complex pattern that leads to oedema disease, where the simple presence of ETEC is not always sufficient to produce clinical disease. It is known as it is also necessary to consider other physiological, environmental, and dietary effects that may sometimes be as important as the ETEC bacteria themselves (33): dietary changes, multisource early weaning, continuous flow of pigs through the facilities, sanitation or respiratory viral disease (34–36). Positive farms usually experience the problem indefinitely, with sporadic periods of apparent improvement (37).

The application of an ED preventive protocol in May 2020 was able to drop again mortality to 3,32%, increasing the production stability to higher levels compared also with Period 1, as expressed by 9 consecutive points under the RBAR of the MR chart and by the reduced frequency of out of control signals (0%). This agrees with Mesonero-Escuredo et al. (30) that observed a significantly higher mortality in Non-vaccinated weaners compared with the vaccinated group, with the risk ratio of dying/being culled for a pig in the Non-vaccinated group around 5 times higher than that of the vaccinated group. Moreover, modifications of feed adopted in the present work (continuous provision of fiber and a reduced protein diet), modulate fimbrial receptors which may be involved in a reduced colonization by *E. coli* after weaning and might decreased production of toxic protein metabolites (38).

Other authors applied SPC charting in livestock as a statistical method for an aggregated analysis from several farms (18, 23), but studies focused on its potential for the single farm when applied in the field are not common (22). This is the first application of SPC charting in a weaning site affected by ED as a monitoring tool of pig health through a key parameter. Two major themes arose in the present study: first, the application of the proposed ED preventive protocol was effective in reducing mortality in the weaning site and the analysis was able to clearly recognize the improvements in the production process. Second, the onset of ED clinical signs severely destabilized the production progress for nearly 4 years until the farmer's decision to apply a rigorous and efficacious ED preventive program. This second observation might reflect the absence of a helpful tool in the field to monitor, revise and implement changes in process or procedures of care with iterative re-evaluation of quality improvement during time. SPC charts might represent the evolution of an older quality improvement tool such as the clinical audits, that are widely described in human medicine and sometimes applied in veterinary practices on companion animals, but scarcely reported in livestock (39). The limitation of clinical audits is that in human medicine they typically rely on comparison of current practice or outcomes to well defined and evidence-based “gold standards”. In veterinary medicine, lack of evidence-based standards in many areas means that clinical audits may be done only to compare practice with a consensus or opinion-based standard, or may be used to create standards or values that allow individual practices to benchmark against processes or outcomes of other practices (40, 41). Moreover, workload, duration, and complexity of case accrual and follow-up are consistently reported as a barrier to clinical audits (42, 43), in particular when applied in veterinary medicine (39). SPC charts might solve some of the main limitations of clinical audits, as one or few basic key parameters [iceberg parameters; (44)] may be selected and used to monitor the productive process of the farm during time compared to own data of the past. For example, mortality as a key parameter was already used by other authors to present statistical control tools for the dynamic monitoring of pig production (45). Retrospective SPC charting may be more challenging to undertake due to the need of a high-quality data collection in the past, that might cause the impossibility to address every single signal of special cause variation in the past (as well as in the present study), but prospective analysis may be easily planned. In practice, the single farm adopting a real-time monitoring of its productive process might be able to early recognize out of control signals, but also to calculate a new PCL and limits when the chart signals a sustained change via the tests (e.g., tests 2 and 3), reflecting a new level of performance. Since the charts are set up with an upper as well as a lower control limit, it is possible to not only give a signal when the process turns in the unfavorable direction, but also to give a positive signal when the process shifts in the desired direction (20), for example after batches 47–49 in present study (positive for test 2). This allow the farm to monitor the own results and shift the own limits even when the method is introduced for the first time in a period that cannot be defined in control, differently from the Period 1 available in the present study. In the specific case of the positive shift suggested at batches 47–49, unfortunately a sudden new increase in mortality appeared from batch 52 showing again an out of control process. In fact, the out of control point may exist without prior alarm or warning signal (46). In a real-time application of SPC charts, considering that every out of control point suggests that a problem impacts the process, it is appropriate to identify the problem and address it. Usually, signals for which no explanation can be found should not be regarded as poor performance by the model, but they rather might denote a problem which was not well realized by the caretaker (47). However, some “false alarm” might occur, for example when the SPC charts produce a signal of special cause variation but no following out of control points appear (46). In this study, several signals of special cause variation preceded one or more out of control points (Figure 1, batches 60 in MR chart; 42–49 in I chart), suggesting that these signals should be considered as “real alarms”. Differently, signals on batches 20–20 should be considered as “false alarms” as they were followed by in control points. However, should be mentioned that the sudden appearance of out of control points showed by this study, often not preceded by warning signals of special cause variation, might be strictly connected with the various and complex pattern that leads to oedema disease. For some situations, one or more signals were found in the I chart but not in the MR chart or vice versa. Thus, the usefulness to construct both the charts with the same data set is the possibility to increase the sensitivity of the SPC approach (27). The application of a SPC monitoring tool in the single farm might totally meet the new frontiers in the livestock management. In fact, recently it has emerged the concept of Precision Livestock Farming (PLF): a holistic approach that adds information and communication technologies to improve the farming process (48). PLF plays an important role in the industrial revolution of livestock, as it uses information and communication technologies to reduce investment costs and increase both production and animal health (49). In traditional livestock farming, decisions are often based only on the experience of the producer. In PLF, such decisions are based on quantitative data of an iceberg or economic parameter, such as mortality or piglets weaned per sow. In addition, quantitative data can be obtained in real-time. To obtain and study such data, in real-time, PLF systems use data analysis, machine learning, control systems, and information and communication technologies (49). To improve efficiency, productivity, livestock nutrition and animal health, it is essential to correctly manage data generated every day in farms (50). Technology over the years has made easier to carry out traditional farm activities. Specifically, in livestock production, it is now possible to process data collected daily related to animal control (51), and SPC might be an adequate tool.

A limitation of study was that special causes in Period 1 were passed and no information or plausible reason could be identified. In some cases, a retrospective enquiry might identify plausible reasons (e.g., breakdown of the heating system in the nursery), but often the lack of registered information is susceptible to bias. Ideally, a Non-biased method is required to identify reasons for unexpected signals, and PLF might help toward this goal. However, this limitation is strongly diminished in case of a real-time and prospective SPC charting. A second limitation was the impossibility to expand the knowledge of SPC charting applied on the monitoring of an ED preventive protocol by an aggregated analysis from several farms as previously showed by other authors (18, 23), that also reported the frequency of signals of special cause variation found during the process. Finally, some drawbacks about SPC charts should be mentioned: first, an alarm does not give information about the problem that caused the signal in the process, and the farmer might be unsatisfied by the impossibility to always track back to the sources of variation, unless a high-quality data collection (15). Second, for some parameters it might be a delay between the change in the system and the time when the observation becomes available. For example, the result of an insemination is not known until a return to estrus or a pregnancy diagnosis is performed. The change in the probability of conception cannot be signaled immediately. Therefore, the specific variability and dynamics of a production system affect the performance of the control chart. In conclusion, the retrospective application of SPC charts in the present study was able to confirm the efficacy of an ED preventive protocol in reducing mortality as a productive iceberg parameter in a piglets weaning site. SPC charting is suggested as a useful tool for the field, other than for research purposes, to provide insights into relationships between health, managerial, and welfare decision and some selected iceberg parameters in livestock. Considering the forthcoming advent of PLF, a prospective application of SPC charting is proposed for the real-time monitoring of the single farm.

## Data Availability Statement

The datasets presented in this study can be found in online repositories. The names of the repository/repositories and accession number(s) can be found below: (Figshare, doi: 10.6084/m9.figshare.17008765).

## Ethics Statement

Ethical review and approval was not required for the animal study because no animals were directly used for experimental purposes, but only data collection about their performances was evaluated and analized.

## Author Contributions

AS, CM, and CC: conceptualization. AS: methodology, formal analysis, and writing—original draft preparation. AS and MF: investigation. AS and PN: writing—review and editing. All authors have read and agreed to the published version of the manuscript.

## Conflict of Interest

MF and CM were employed by Swivet Research sas. CC was employed by Chemifarma spa. The remaining authors declare that the research was conducted in the absence of any commercial or financial relationships that could be construed as a potential conflict of interest.

## Publisher's Note

All claims expressed in this article are solely those of the authors and do not necessarily represent those of their affiliated organizations, or those of the publisher, the editors and the reviewers. Any product that may be evaluated in this article, or claim that may be made by its manufacturer, is not guaranteed or endorsed by the publisher.
